# Five-year tuberculosis trends analysis in eight districts of Mwanza region, Tanzania; (2017–2021)

**DOI:** 10.1186/s12889-024-20684-6

**Published:** 2024-11-19

**Authors:** Medard Beyanga, Novel N. Chegou, Gerhard Walzl, Stephen E. Mshana, Kasang Christa

**Affiliations:** 1 National Public Health Laboratory Ministry of Health, Mabibo, Dar es Salaam, Tanzania; 2 South African Medical Research Council Centre for Tuberculosis Research, Division of Immunology, Department of Biomedical Sciences, Faculty of Medicine and Health Sciences, Stellenbosch University, P.O. Box 241, Cape Town, 8000 South Africa; 3 Department of Microbiology and Immunology, Weill Bugando School of Medicine, Catholic University of Health and Allied Sciences, P.O. Box 1464, Mwanza, Tanzania; 4 German Leprosy and TB Relief Association, Wurzburg, Germany

**Keywords:** Tuberculosis, Trends, Detection, Rate

## Abstract

**Background:**

In Tanzania like other developing countries, TB detection is hindered by totally missed, late notification, and delayed diagnosis of active cases. Apart from having TB control strategies and interventions to detect patients and put them on treatment to cut down the chain of transmission, TB remains a health concern. Limited data exist on the burden and trends of tuberculosis in Mwanza, which includes fishing communities and living conditions that are associated with high TB transmission like overcrowding. This study aimed to determine tuberculosis trends in the Mwanza region of Tanzania for five years, from 2017 to 2021.

**Methods:**

We extracted routine TB diagnostic data from 2017 to 2021 from eight districts of the Mwanza region of Tanzania from the electronic TB database. Data were captured in Microsoft Office Excel 2007 with district TB and leprosy coordinators and then imported into STATA 13 (Stata Corp LLC, College Station, TX, USA) for analysis. We estimated the TB case detection rate per 100,000 population.

**Results:**

A total of 6,414 laboratory-confirmed tuberculosis cases were detected in eight districts of the Mwanza region in Tanzania from 2017 to 2021. The average tuberculosis detection rate in five years was 34.7 per 100,000 population. Overall, the TB detection rate was two times higher in people without HIV (30.5) compared to those infected with HIV; 13.4 per 100,000 population. Of the 15 rifampicin-resistant TB cases detected in the year 2018, 66.7% (10/15) were HIV-negative compared to 33.3% (5/15) infected with HIV.

**Conclusion:**

The TB case detection rate decreased in Mwanza region from 43.9 in 2017 to 21.4 per 100,000 population in 2021. Other parameters were missing in the database, which indicates remarkable gaps in the established database to monitor TB management in the region. The program may consider investigating and improving the documentation of information necessary to attain its goals.

## Introduction

Tuberculosis continues to infect people and cause death globally, despite the availability of effective drugs to cure the disease [1]. In the year 2022, TB was estimated to cause about 1.3 million deaths worldwide [2]. TB incidence rate (new cases per 100,000 population per year) reduced to 2% between 2010 and 2020. However, due to the COVID-19 pandemic, the worldwide incidence rate increased to 3.6% between 2020 and 2022 pulling back the global effort to end TB by 2030 [2]. In Tanzania, the TB incidence rate reduced by 27% from 306 in 2015 to 222 per 100,000 population per year in 2020, and TB notification decreased to 146 from 148 per 100,000 population between 2020 and 2021 [3]. Like many other African countries, the most limiting factors for TB notification continues to be totally missed, late notification and delayed diagnosis of TB active cases [4]. During the study period, the region had a total of 10 GeneXpert instruments, one at each district laboratory but sputum smear microscopy remains the most used method to diagnose the disease leaves about 37% of patients undiagnosed because of its poor sensitivity [5] Therefore, programs are working with unrealistic burden estimates because cases remain undetected and where detection is done, documentation is inefficient to report all cases accurately [6]. Limited data exist on the burden and trends of tuberculosis in Mwanza, which includes fishing communities and living conditions that are associated with high TB transmission like overcrowding. Apart from having TB control strategies and interventions to detect patients and put them on treatment to cut down the chain of transmission, TB remains a health concern. Understanding the disease trends is critical to guide stakeholders to identify outstanding problems and also assess the effectiveness of the interventions applied to control the disease. Therefore, it is necessary to provide updated information regarding the TB detection rate to the National TB and Leprosy Control Program (NTLP) as results from trend analysis have been useful in describing the disease patterns, comparing disease time periods and propose feature intervention for disease prevention and control [7]. Five –year trend analysis of TB in Mwanza region is the first attempt and is critical to understand the burden and detection rates, especially during COVID-19 pandemic so that NTLP can be informed accordingly. Therefore, the present study aims at determining the trends of tuberculosis in Mwanza region Tanzania for a period of five years, from 2017 to 2022.

## Methods

### Study setting and period of the study

Mwanza region shares a boundary with five regions and is occupied by fishing and peasant populations. It borders Simiyu to the east, Geita to the west, Shinyanga to the south and on the Lake Victoria is bordering Kagera and Mara. Mwanza is the second biggest region in Tanzania after Dar es Salaam having a population of 3,699,872 people as per 2022 National census [8, 9]. The region occupies an area of 25,233 square kilometers where by 9,743 Km^2^ is water and 11,796 Km^2^ is dry land [10]. Eight districts of Mwanza region with their population are Buchosa (413,110), Ilemela (509,687), Kwimba (480,025) and Magu (421,119). Other districts include Misungwi (467,867), Nyamagana (594,834), Sengerema (425,415) and Ukerewe (387,815) [8]. Like other regions in Tanzania, TB notification, treatment, prevention and control is overseen by the district TB and leprosy coordinators (DTLCs). The coordinators supervise health centers and primary health care facilities where TB patients attend like other normal patients. Each district hospital and health center has a designated TB clinic where TB patients are notified, diagnosed and managed using pre-trained health care workers. Each TB clinic is assigned by personnel to serve as a direct observed therapy short course (DOTS) site and carry out community surveillance of the disease, monitor treatments and traces lost follow up patients. Other tasks carried out by a DOTS nurse includes collaborative community engagement and forms linkage between Partners and Government in his or her area of work. DTLCs report to the Regional Medical Officer (RMO) all issues regarding the TB program, then to the National TB and Leprosy control program. The trends analysis study was carried out in December 2022.

### Study design

This is a retrospective review of TB data collected as routine notification, diagnosis and treatment of TB patients as per Tanzania National TB and leprosy control program (NTLP). Data were extracted from the National TB database on TB indicators.

### Data collection and analysis

We extracted TB case data from an electronic database developed to manage routine data collected for five years, from 2017 to 2021, for the Mwanza region. Data were cleaned and transferred into Microsoft office Excel 2007 in collaboration with district TB and leprosy coordinators (DTLCs) under the supervision of the regional TB and leprosy coordinators (RTLCs). Data were then imported into STATA 13 (Stata corp LLC, College Station, TX, USA) for analysis. TB trends analysis were reported as simple descriptive data using tables and graphs. The detection rate was estimated based on per 100,000 population using data sources available at citypopulation.de/en/Tanzania/Admin/19_mwanza.

### Case definitions

Tuberculosis positive case (TB+) as used in the current study implies any person who was microbiologically confirmed to have TB by smear microscopy, culture or xpert MTB/RIF assay from at least one specimen [11]. Rifampicin resistant tuberculosis (RR-TB) were reported by Xpert MTB/RIF.

### Ethical considerations

The informed consent from all participants was deemed not necessary by the joint Catholic University of Health and Allied Sciences (CUHAS)/Bugando Medical Centre (BMC) Research Ethics and Review Committee (CREC) (Number CREC/398/2019) and the Health Research Ethics Committee of Stellenbosch University, Cape Town, South Africa (reference number: S21/5/081). Permission for access to the TB program database was obtained from the National TB and Leprosy control program manager, through the Ministry of Health.

## Results

**Fig. 1:**
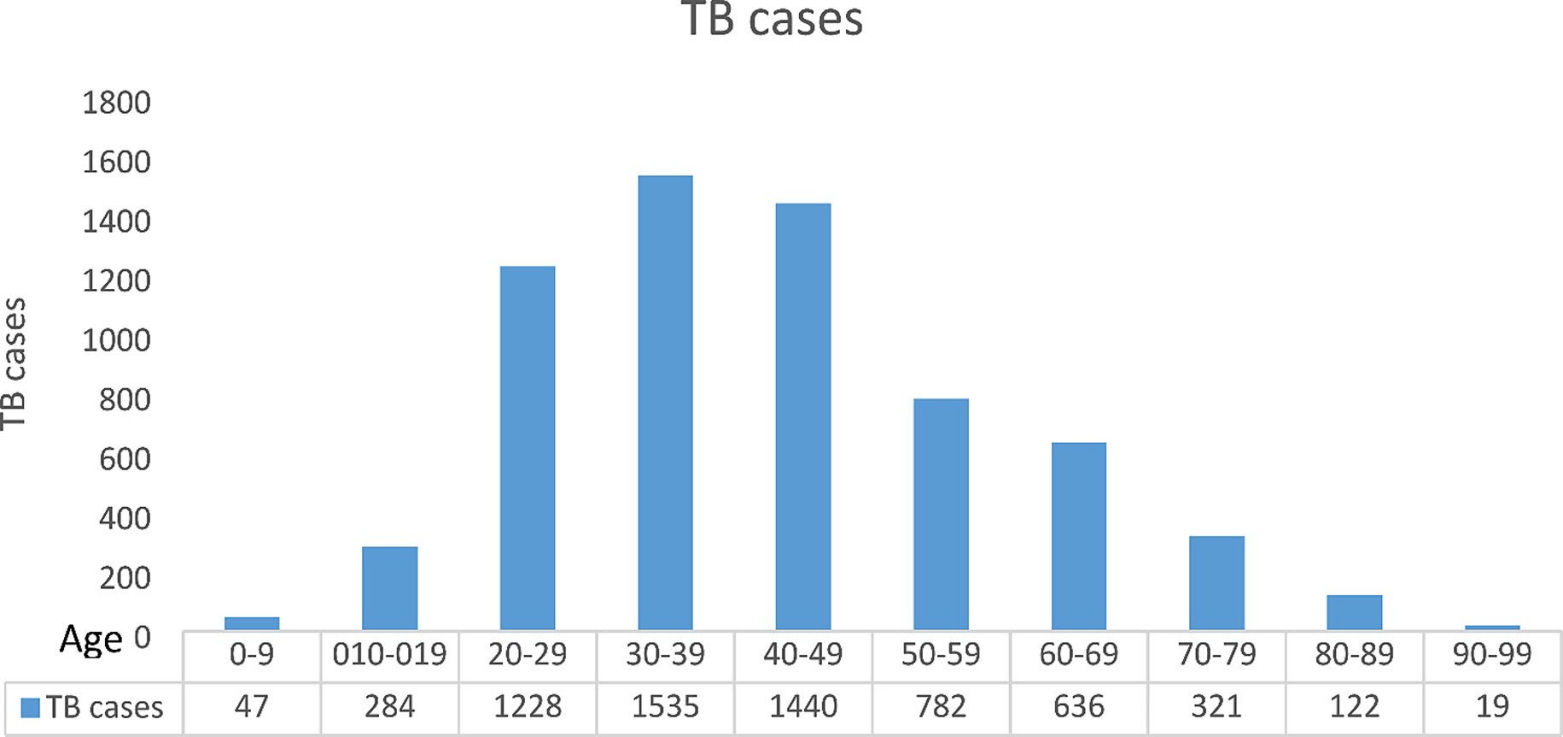
Distribution of TB cases in different age groups in eight districts of the Mwanza region, Tanzania from 2017 to 2021

A total of 6,414 laboratory confirmed tuberculosis cases were detected in the eight districts of Mwanza region in Tanzania during the year 2017 to 2021. Most cases (*n* = 4186; 65.3%) were females (Table 1). Of all the TB cases that were detected during the study period, 0.7% (47) were below 9 years, 4.4% (284) between the ages of 10 and 19years, 65.5% (4203) were between the ages of 20 and 49 years and 29.3% (1,880) ≥ 50 years, up to 99 years **(**Fig. 1**)**.

Regarding the method used for diagnosing TB in the cases, majority (66%) *n* = 3875 were detected using smear microscopy and (34%) *N* = 2539 by geneXpert MTB/RIF. Average tuberculosis detection rate in five years (2017 to 2021) were 34.7 per 100,000 population compared to the National detection rate of 37.82 in 2015 and 65.65/ 100.000 population in 2021. We observed a 2-fold annual decline in the TB detection rate, from 43.9 cases in 2017, to 21.4 cases per 100,000 population in 2021.

More than half (71.7%, *n* = 4596) of the TB cases detected were HIV negative and 0.23% (15/6414) cases were TB rifampicin-resistant (RR-TB) **(**Table 4**).** In 2018, Magu and Nyamagana districts had high detection rate compared to other districts 58.4 and 84.2 per 100,000 population respectively **(**Table 2**).** Of the 15 patients that were diagnosed with rifampicin resistant TB (RR-TB) during the study period, 66.7% (10/15) were uninfected with HIV and 33.3% (5/15) of cases were detected in year 2018. Among laboratory reported TB cases, 6365 cases were classified as pulmonary, 32 extra pulmonary and 17 cases were classified as both pulmonary and extra pulmonary tuberculosis.

**Fig. 2:**
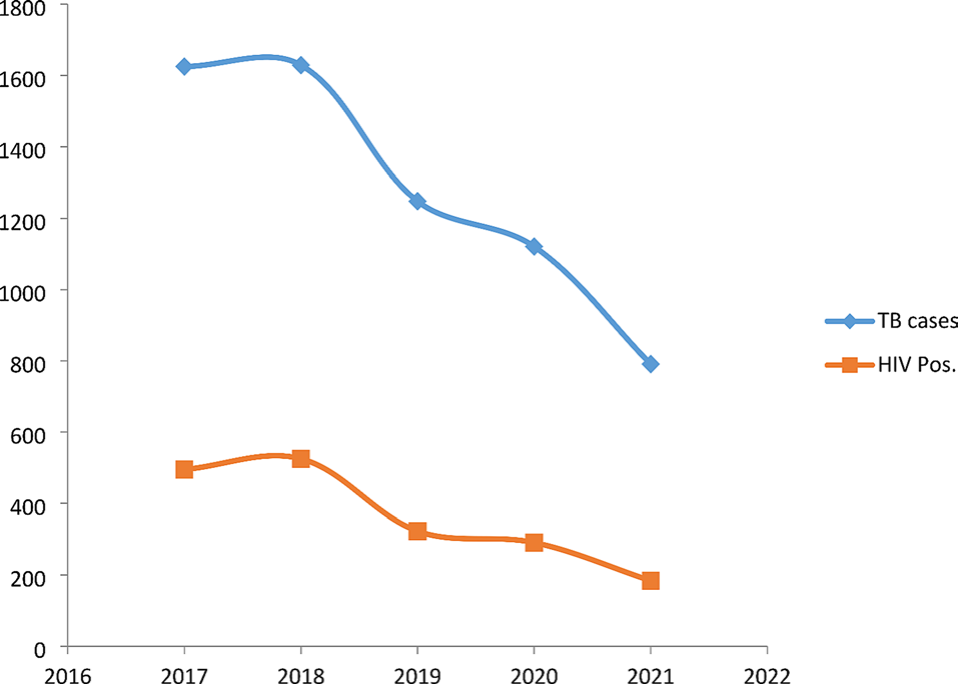
Trends of TB case detection in eight districts of the Mwanza region, Tanzania, from 2017 to 2021. The figure presents data on annual TB detection rate (blue line) and HIV-positive individuals separately (red line) during the period of study

**Table 1:** TB cases detected in eight districts of Mwanza Region Tanzania (2017–2021)

Characteristics	Total *n* = 6414	HIV Pos. *n* = 1818	HIV Neg. *n* = 4596
**Year**			
2017	1625 (25.3)	495 (27.2)	1130 (24.6)
2018	1629 (25.4)	525 (28.9)	1104 (24.0)
2019	1248 (19.5)	323 (17.8)	925 (20.0)
2020	1121 (17.5)	291 (16.0)	830 (18.1)
2021	791 (12.3)	184 (10.1)	607 (14.6)
**Districts**			
Buchosa	663 (10.3)	230 (12.6)	433 (9.4)
Ilemela	556 (8.7)	151 (8.3)	405 (8.8)
Kwimba	432 (6.7)	105 (5.8)	327 (7.1)
Magu	998 (15.6)	327 (18.0)	671 (14.5)
Misungwi	691 (10.8)	158 (8.6)	533 (11.5)
Nyamagana	1840 (28.7)	506 (27.8)	1334 (29.0)
Sengerema	896 (14.0)	263 (14.5)	633 (13.7)
Ukerewe	338 (5.3)	78 (4.3)	260 (5.7)
**Sex**			
Male	2228(34.7)	772(42.5)	1456 (31.7)
Female	4186(65.3)	1046(57.5)	3140 (36.8
Rifampicin resistance			
RR-TB	15 (0.2)	5(0.02)	10 (0.2)
% diagnosed			
Smear microscopy 34% (*n* = 2539)			
Xpert 66% (*n* = 3875)			

**Table 2:** Trends of overall TB case detection (CDR) in eight districts in Mwanza Tanzania (2017–2021) RR-TB, rifampicin resistance tuberculosis;

Years	Total case detection rate (CDR) per 100,000 population				
	2017	2018	2019	2020	2021
Overall	43.9	44	33.7	30.3	21.4
**Districts**					
Buchosa	19.3	40.9	29.3	30.3	40.7
Ilemela	15.7	23.9	20.4	18.6	12.8
Kwimba	16.7	17.3	18.5	8.7	21.7
Magu	18.9	58.4	45.8	49.7	25.9
Misungwi	17.1	40.2	26.5	34.8	18.6
Nyamagana	13.4	84.2	68.3	55.8	17.1
Sengerema	18.8	56.9	34.1	26.3	22
Ukerewe	20.6	20.1	17	10.8	15.9
**Sex**					
Male	32.3	31	24.1	20.5	15.6
Female	54.9	56.4	42.8	39.6	26.8

**Table 3:** Trends of overall TB CDR among HIV infection subgroups in eight districts of Mwanza Tanzania (2017–2021)

Year	CDR among people with HIV per 100,000 population					CDR among people without HIV per 100,000 population				
	2017	2018	2019	2020	2021	2017	2018	2019	2020	2021
Overall	13.4	14.2	8.7	7.9	5	30.5	29.8	25	22.4	16.4
**Districts**										
Buchosa	5.1	16.9	10.4	10.7	12.6	14.3	24	18.8	19.6	28.1
Ilemela	10.8	7.8	2.9	4.3	3.7	22.6	16.1	17.5	14.3	9
Kwimba	6.2	4.8	5	3.1	2.7	17.5	12.5	13.5	5.6	18.9
Magu	20.9	22.1	13.8	16.1	4.7	36.1	36.3	32.1	33.7	21.1
Misungwi	8.1	8.1	5.1	7.1	5.3	19.4	32.1	21.4	28	13.3
Nyamagana	24	26.7	17.8	13.1	3.4	59.8	57.5	50.4	42.7	13.8
Sengerema	23	18.8	9.6	4.7	5.6	48.2	38.1	24.4	21.6	16.5
Ukerewe	5.7	5.7	3.1	2.8	2.8	17.5	14.4	13.9	8	13.2
**Sex**										
Female	12.6	12.2	7.8	6.2	4.2	19.6	18.9	16.4	14.3	11.5
Male	14.1	16.1	9.6	9.5	5.7	40.8	40.3	33.2	30.1	21.1

**Table 4:** TB case detection among HIV positive and negative patients in the Mwanza region from 2017–2021

	Case detection among HIV +					Case detection among HIV –				
	2017	2018	2019	2020	2021	2017	2018	2019	2020	2021
Overall	495	525	323	291	184	1130	1104	925	830	607
**Districts**										
Buchosa	21	70	43	44	52	59	99	78	81	116
Ilemela	55	40	15	22	19	115	82	89	73	46
Kwimba	30	23	24	15	13	84	60	65	27	91
Magu	88	93	58	68	20	152	153	135	142	89
Misungwi	38	38	24	33	25	91	150	100	130	62
Nyamagana	143	159	106	78	20	356	342	300	254	82
Sengerema	98	80	41	20	24	205	162	104	92	70
Ukerewe	22	22	12	11	11	68	56	54	31	51
**Sex**										
Female	227	219	140	111	75	356	340	295	258	207
Male	268	306	183	180	109	774	764	630	572	400
RR-TB	1	2	0	2	0	1	7	1	0	1

## Discussion

In the current study, we analyzed routine data collected from tuberculosis patient management from 2017 to 2021. During the analysis, we found an average TB case detection rate of 34.7 cases per 100,000 populations in a period of five years (2017–2021).

Our study is similar to what was studied in Uganda’s rural area involving eight districts which reported an average of 149 cases per 100,000 population in five years (2015–2019), However, the two findings can’t be compared because of different study periods [12]. There was a significant decline in the annual tuberculosis detection rate (Fig. 2) in the Mwanza region from 1625 cases in 2017 to 791 cases in 2021. Despite a decline in TB notification trends in the Mwanza region, this was not the case in the individual districts, for example in the Buchosa district, the detection rate increased in 2021 after the installation of the GeneXpert instrument. However, the COVID-19 pandemic could have contributed to a decrease in TB detection due to the diversion of health care services towards COVID-19 challenges and disruption of the TB care continuum. However, the decline in cases was already evident before the COVID-19 pandemic and the fewer data points available for pre and post-COVID-19 analysis preclude the performance of a regression model. Tanzania did not go for complete lockdown during the COVID-19 pandemic. However, several services supporting TB case diagnosis were interrupted and this hindered TB detection supplies from reaching the testing facilities, for example supply of GeneXpert for TB detection [13]. Adults ranging from 20-60years accounted for 78.6% of all cases, this observation is quite similar to what was observed in a five-year tuberculosis trends analysis conducted in Awi Zone, Northwest Ethiopia which found almost the same age group accounting for 87.3% of all TB cases [14]. The smear microscope diagnostic method detected 66.0% of all TB cases meaning that in the absence of GeneXpert MTB/RIF, 34% percent of additional cases could have remained undiagnosed, the major limitation to reach the target of stopping TB by 2030. It has been documented that GeneXpert MTB/RIF increases TB detection rate by up to 47% compared to smear microscopy [15]. Therefore, adding and expanding access to geneXpert MTB/RIF technology could significantly increase the TB detection rate. TB detection rates were high 65.3% in men compared to women in the Mwanza region. This finding complements what has been strongly reported by a systematic review highlighting that TB is high in men because men do not benefit from accessing TB care in most cases. The report recommends specific TB program strategies to improve men’s access to tuberculosis care [16–18]. The TB detection rate was high in people without HIV 71.7% (4596) and 0.23% (15/6414) cases were tuberculosis rifampicin resistance (RR) **(**Table 3**).** We argue that this is due to strengthened HIV prevention and control response and enhanced TB/HIV collaborative activities. Countries and regions with high burdens of HIV and TB strengthen and sustain efforts to achieve the goal of ending both HIV and TB epidemics in line with the Sustainable “Development Goals [19].

Our findings on RR-TB of 0.23% in the Mwanza region were below the pooled prevalence of RR-TB reported by a systematic review that synthesized evidence on the prevalence of RR-TB in East Africa. The results of this meta-analysis survey reported the pooled prevalence of newly diagnosed RR-TB to 4% (95% CI 2–5%) [20]. Ethiopia reported rifampicin-resistance of 8.73%, almost doubling that of Uganda.

## Study limitation

The data analyzed were from the TB database and were retrospectively collected. There was missing critical information that could have added more value to our publication, a major limitation of the study. Variables including treatment success rate and their associated factors were missing. We couldn’t look at actual incidence-to-notification ratios this would have been a gold standard in understanding if the TB burden is going down or if this change is a reflection of the healthcare system.

## Conclusion

The TB case detection rate decreased in the Mwanza region from 2017 to 2021. Other parameters were missing in the database which indicates remarkable gaps in the established database to monitor tuberculosis management in the region. The program may consider investigating and improving the documentation of information necessary to attain its goals.

## Data Availability

Data of this study is available and can be shared once requested. In case data for this study is needed pleases contact the corresponding author of this paper.

## Abbreviations

BMC: Bugando medical centre
CUHAS: Catholic university of health and allied sciences
CDR: Case detection rates
CREC: Clinical research ethical committee
DOTs: Direct observed treatments
DTLCs: District tuberculosis and leprosy coordinator’s
RMO: Regional medical officers
NTLP: National tuberculosis and leprosy program
RR: Rifampicin resistance
RTLCs: Regional tuberculosis and leprosy coordinator’s
